# Interventions to Improve Social Climate in Acute Mental Health Inpatient Settings: Systematic Review of Content and Outcomes

**DOI:** 10.1177/23779608221124291

**Published:** 2022-12-12

**Authors:** Geoffrey L. Dickens, Alisha Johnson, Kelly Steel, Bronwyn Everett, Matthew Tonkin

**Affiliations:** 1 Centre for Applied Nursing Research, South Western Sydney Local Health District and 6489 Western Sydney University, Ingham Institute for Medical Research, Liverpool, NSW, Australia; 2 South Western Sydney Local Health District Mental Health Service, Liverpool Hospital, Sydney, NSW, Australia; 3 School of Nursing and Midwifery, 6489 Western Sydney University, Parramatta, NSW, Australia; 4 School of Criminology, 4488 University of Leicester, Leicester, UK

**Keywords:** health facility environments, mentally ill persons, systematic review, therapeutic climate, ward climate

## Abstract

**Introduction:**

Quantification of the social climate of mental health care environments has received considerable attention. Investigations of the resulting measures indicate that social climate is associated with individual outcomes including patient satisfaction and staff burnout. Interest has grown in developing interventions to improve social climate in anticipation of subsequent related benefits. This study aimed to identify and critically review research about the effectiveness of interventions for improving social climate in inpatient adult acute mental health settings.

**Methods:**

Systematic review reported in line with the Preferred Reporting Items for Systematic reviews and Meta-Analyses. Comprehensive terms were used to search multiple electronic databases from inception to July 2019. Information about intervention type(s), complexity was extracted and study quality was assessed.

**Results:**

Twenty-three papers met inclusion criteria of which 20 used a pretest–posttest study design and three employed randomized and/or controlled designs. Interventions were environmental/structural, operational/process-oriented and developmental/person-oriented in nature and they ranged in complexity. The Ward Atmosphere Scale was the most common outcome measure used. Following quality assessment, six studies were judged to be sufficiently robust in terms of quality, theory-base, user-inclusion, and outcomes evaluation to contribute credibly to the evidence base. Of these, four complex person- and process-oriented intervention studies and two less complex structural/environmental intervention studies resulted in positive outcomes.

**Conclusion:**

There is limited strong evidence that interventions positively influence measures of ward social climate in acute adult mental health settings. Such measures should not be the sole criterion of success when evaluating change. Decisions about implementing change to improve social climate should be informed by meaningful proxy measures including the views and preferences of service users and other stakeholders. Studies using stronger designs are required to establish the ability of interventions to improve social climate.

## Introduction

In healthcare settings, it has long been held axiomatic that a range of staff, patient, organizational and environmental factors contribute toward the overall ward or organizational climate (Moos, 1986; Schalast et al., 2008; World Health Organisation, 1953), also termed the ward culture, atmosphere, or milieu. In turn, it has been proposed that ward climate is a determinant of important indicators of organizational health including staff well-being, satisfaction, burnout, retention, turnover, and user autonomy, patient treatment outcome and safety (Caldwell et al., 2006; Hendel, 1993; Stone et al., 2006; Timko, 1996; Tumulty et al., 1994). The various constructs appear to overlap and there is a lack of distinction between them (Duxbury et al., 2006). Definitions using the term “climate” tend to emphasize the contribution of human relationships (Ekvall & Ryhammar, 1998), while “atmosphere” and “milieu” may place more emphasis on the interaction between people and the physical environment (Nicholls et al., 2015). However, the distinction is not clear: for example, the two measurements reported most frequently in mental health settings, the Ward Atmosphere Scale (Moos, 1986) and the Essen Climate Evaluation Schema (Schalast & Groenewald, 2009; Schalast et al., 2008) largely draw on ratings of how people perceive their surroundings based on interactional elements. In this study, we use the term “climate” though we intend the term to apply to any broadly similar construct.

In inpatient mental health settings, ward climate is considered to be of special importance because the social context in which therapy and treatment are delivered is thought to be a key contributor to its success. Almost 70 years ago, the World Health Organization described the ward atmosphere as “the single most important factor in treatment efficacy” in inpatient mental health services, despite describing the construct as “intangible” (World Health Organisation, 1953: 17). Reflecting this, researchers have aimed to develop valid and reliable measures of ward climate and to investigate the relationships between those measures and real-world outcomes such as patient satisfaction, patient symptomatology (Eklund & Hansson, 1997, 2001; Jorgensen et al., 2009), length of stay, level of functioning (Melle et al., 1996), and patient aggression (Isaak et al., 2017). Results have indicated associations between favorably perceived environments and positive individual outcomes. While not demonstrating causal direction, these results suggest a theoretical potential to improve individual outcomes by intervening to improve the ward climate. Surprisingly, little research has examined whether such interventions work *on their own terms:* that is, do they demonstrably change the measured climate. Such a question is likely to be of considerable interest to nurses who may be considered to contribute disproportionately to the social climate of inpatient wards since they are the only professional group who are present around the clock and thus best placed to promote interventions to improve matters.

In the above context, the aim of the current study was to appraise the evidence about the effectiveness of interventions in adult, acute mental health wards for improving social climate as measured using social climate scales. The review question therefore is: are interventions to improve ward climate effective in improving measured climate irrespective of how the studies have chosen to define and operationalize climate? Specific objectives were to identify which interventions show the most promise in terms of improving social climate and determine whether they share characteristics in terms of their type and complexity.

## Methods

The review is reported in accordance with the Preferred Reporting Items for Systematic Reviews and Meta-Analyses statement (Moher et al., 2009).

### Search Methods

The aim was to identify accounts of primary research that investigated the effectiveness of an intervention for improving ward climate in acute mental health inpatient settings. The outcome of interest was the construct of climate itself as measured using an appropriate climate-related scale. The two main criteria for study inclusion, therefore, were (i) that there had been a planned intervention one of whose implicit or explicit aims was to improve the ward climate; and (ii) that at least one of the outcome measures utilized was designed to capture any resulting change in climate. The search was conducted in two stages.

#### Stage I: Scale identification

A range of relevant literature reviews was consulted (Colla et al., 2005; Gershon et al., 2004; Jung et al., 2009; Scott et al., 2003; Singla et al., 2006; Tonkin, 2016) and supplemented with personal knowledge, discussion with expert colleagues, and searches of the Scopus database for relevant scale development papers. As a result, 16 tools were identified for inclusion in part two of the literature search (see Table 1).

**Table 1.** Climate Measurement Tools Included in Literature Search.

Ward Atmosphere Scale (Moos, 1986; Moos & Houts, 1968) Essen Climate Evaluation Schema (EssenCES) (Schalast & Groenewald, 2009; Schalast et al., 2008) Creative Climate Questionnaire (CCQ) (Ekvall et al., 1983) E13 (Bjorkdahl et al., 2013) Organizational Culture Assessment Inventory (OCAI) (Cameron & Quinn, 1999) SOCRaTEs: A measure of the Social Climate in Therapeutic Environments (Clarke & Freestone, 2013) Residential Substance abuse and Psychiatric Programs Inventory (RESPPI) (Timko, 1994, 1995) Good Milieu Index (GMI) (Friis, 1986) Community Oriented Programs Environment Scale (COPES) (Moos, 2009) Violence Prevention Climate-14 (VPC-14) (Hallett et al., 2018) Safety Attitudes Questionnaire (SAQ) (Sexton et al., 2006) Veterans Health Administration Patient Safety Questionnaire (Singla et al., 2006) Hospital Safety Culture Questionnaire (HCSQ) (Itoh et al., 2002) Safety Climate Survey (SCS) (Landesman & McKnight, 2006) Teamwork and Patient Safety Attitudes Questionnaire (TPSAQ) (Kaissi et al., 2003) Views on the Therapeutic Environment measure (VOTE) (Laker et al., 2012)

**Table 2.** Example Electronic Database Search (CINAHL).

	Search terms	Limiters
Population Intervention Comparator Outcome	((Mental health OR psychiatry**) AND (Inpatient OR in-patient OR ward OR hospital OR institution OR asylum OR organisation OR organization)) AND No limit AND No limit AND ((Ward Atmosphere Scale OR WAS) AND (Moos)) OR ((Essen Climate Evaluation Schema OR EssenCES) AND (Schalast)) OR ((Creative Climate Questionnaire OR CCQ) AND (Ekvall)) OR ((E13) AND (Bjorkdahl)) OR ((Organizational Culture Assessment Inventory OR OCAI) AND (Cameron OR Quinn)) OR ((SOCRaTEs OR Social Climate in Therapeutic Environments) AND (Clarke OR Freestone)) OR ((Residential Substance abuse and Psychiatric Programs Inventory OR RESPPI) AND (Timko)) OR ((Good Milieu Index) OR GMI) AND (Friis)) AND ((Community Oriented Programs Environment Scale OR COPES) AND (Moos)) OR (((Violence Prevention Climate-14) OR VPC-14) AND (Hallett)) OR ((Safety Attitudes Questionnaire) OR (SAQ) AND (Sexton)) OR ((Veterans Health Administration Patient Safety Questionnaire) AND (Landesman)) OR ((Hospital Safety Culture Questionnaire) OR (HSCQ) AND (Itoh)) OR ((Agency for Healthcare Research and Quality Hospital Survey on Patient Safety Questionnaire)) OR ((Safety Climate Survey) OR (SCS) AND (Sexton)) OR ((Teamwork and Patient Safety Attitudes Questionnaire) OR (TPSAQ) AND (Kaissi))	English Language No date limiter Acute general inpatient mental health only NOT forensic, secure services Not adolescent units, therapeutic communities, elderly or geriatric units

#### Stage II: Main search

To address the review question, a Population-Intervention-Comparator-Outcome (PICO; Schardt et al., 2007) statement was devised and comprehensive search terms related to the key elements were generated (see Table 2 for example search). The population of interest were staff and/or patients working/residing on acute, adult, mental health inpatient wards; the intervention could be any for which a stated or implied aim was to change culture or climate; possible comparators were self (i.e., pretest–posttest studies), treatment as usual (e.g., control ward/s), or another intervention; outcomes were any of the measures identified in stage one of the literature search. Databases searched were CINAHL Medline, Scopus, WorldCat Dissertations and Theses, Google Scholar, and PsycINFO. Reference lists of relevant included studies and literature reviews located during the search were also searched. No date of publication restriction was applied to the searches within these databases. The title and abstract of all articles returned from the search strategy were reviewed by Authors 2 and 3, and the full text of any potentially meeting inclusion criteria was obtained. Eligibility of the full-text studies was reviewed independently by authors 1, 2, and 3 and with minor discrepancies discussed and resolved by the study team.

### Inclusion/Exclusion Criteria

For inclusion in the narrative review, studies must have included data from a minimum of two iterations (pre- and post-intervention) of one of the tools identified in part (i) of the search strategy. Data must have been collected from staff and/or patients working/resident in the study setting. Exclusion criteria were: non-English language studies; studies conducted in nonadult or nonacute mental health settings; longitudinal studies with no intervention; and studies using outcome measures other than those identified in stage I. Identification of any previously nonincluded outcome measures during this stage led to consideration for inclusion. This led to the inclusion of the VOTE (Laker et al., 2012).

### Study Bias

A quality analysis was undertaken by three reviewers (1, 2, and 3) using the Effective Public Health Practice Project's Quality Assessment Tool for Quantitative Studies (Effective Public Health Practice Project, 1998) (see Supplementary Table S1), a tool suitable for assessing studies using a range of quantitative designs.

### Data Extraction

The following data were extracted from included studies: author(s), country, setting, description of the intervention, direct recipients of the intervention, information regarding the proposed theoretical basis of the intervention, study design, climate, and any other measures utilized, participants, and outcomes on climate and other measures (see Table 3). Additionally, we rated the complexity of study interventions according to criteria suggested by Mills et al., (2019): (i) the extent to which it comprises multiple interacting components; (ii) the range, difficulty, and variability of behaviors required by those delivering or receiving the intervention; (iii) the extent to which multiple groups or organizational levels are targeted; (iv) the number and variability of outcomes measured; and (v) the degree of flexibility permitted. Each intervention was rated in each area as lacking complexity, somewhat complex, or definitely complex. An overall complexity rating of complex (definite complexity in three or more of five domains), somewhat complex (somewhat or definitely complex in three or more of five domains), or low complexity (low complexity in three of five domains) was assigned. We also assigned interventions to a category according to their type based on criteria described by Vera and Kuntz (2007): process-oriented or operational changes (e.g., systems or operation redesign), person-centered changes (usually involving staff and aiming to change practice through educational techniques or transformational change through exercises such as group clinical supervision) and structural or environmental change (i.e., hard, physical change to the care environment including new buildings and interiors). Study interventions could be assigned to one, two, or three categories because we examined their constituent parts.

**Table 3.** Study Characteristics.

Study	Setting	Intervention				Implementation and fidelity	Outcomes
		Target	Indicative content	Theoretical basis	Type/complexity		
Aubry et al. (1996)	Canada 21-bed inpatient mental health unit	Staff, patients Total *N* = 89 (66.3% patients)	**1. Needs assessment and actions based on social climate data: *(a). Policy review***: liberalization generally; (***b). New treatment programs***: discharge planning, stress management, health promotion, problem-solving, life skills; (***c) Communication review*:** orientation brochure, daily community meeting, focused care plans **(d) *Staff education:*** staff meetings for discussion. **2. Relocation*:*** From 25-bed to 21-bed new build unit with improved facilities	Empirical links between social climate and patient outcomes.	**Type:** Process, Structural, Person-oriented **Complexity:** Definite	**Implementation:** Recommendations for change made by a multidisciplinary committee of staff Delivery by staff team with special responsibilities assigned to those with expertise. Relocation decided a priori **Fidelity arrangements:** No information	**Design:** Pretest–posttest (follow-up 12-month) **Climate measure:** Ward Atmosphere Scale “Real” version (WAS-R; Moos, 1989) **Results:** Patients: positive impact on: Support (Sup), Spontaneity (Sup), Autonomy (Aut), Practical Orientation (PO), Anger and Aggression (AA), Order and Organisation (OO), Practical Orientation (PO). Staff: Improvement on Program Clarity (PC)
Baumgardt et al. (2019)	Germany Two locked wards	Staff, patients *N* = 168 (47.6% patients)	**Safewards interventions: (**1) mutually agreed behavior (2) short public statements on handling flashpoints, (3) de-escalation model (4) say something good about each patient per shift (5) bad news mitigation(6) shared, personal staff/patient information (7) a regular patient/staff meeting (8) distraction and sensory tools; (9) reassuring explanations following frightening incidents; (10) display of positive messages from discharged patients	Safeward Model (Bowers et al., 2015).	**Type:** Process, Person-oriented **Complexity:** Somewhat	**Implementation:** Overseen by consultant psychiatrists and ward managers. Supervision by chief psychiatrist, chief nurse, and researchers. Standardized all-day workshop attended by all staff. Assignment of Safewards “champions”. One intervention per month over 10 months. **Fidelity arrangements:** Fidelity scores high in both wards.	**Design:** Pretest–posttest (follow-up 12-month) **Climate measure:** EssenCES (Schalast et al., 2008) **Results:** Improvements for staff on Therapeutic Hold (TH), Patient Cohesion (PC), Subjective Safety (SS); improvements; for patients on PC on one ward only (Baumgardt, personal correspondence) **Other results:** Decline in use of coercive measures.
Berg & Hallberg (1999)	Sweden 16-bed ward (psychosis and borderline personality disorder)	Nursing staff *N* = 22	**Systematic clinical group supervision*:*** Case-focused, and discussion on transference and their own related behavior, the patient's problem, the quality of their relationship. **Group supervised nursing care planning:** The areas covered the patient's activities of daily living	Nursing diagnoses (Carnevali & Patrick, 1986); group supervision (Hallberg et al., 1993)	**Type:** Person-oriented **Complexity:** Somewhat	**Implementation:** 1. Introduction day and two FUs within 12-month; 2-weekly 3-h group (66-h total) 2. Weekly 2-h per group (80-h per group total). Author 1 supervised and facilitated groups. **Fidelity arrangements:** No information	**Design:** Pretest–posttest (follow-up 6 and 12-month) **Climate: measure:** Creative Climate Questionnaire (CCQ; Ekvall et al., 1983) **Results**: CCQ: Subscale improvements: Trust, Conflict, Idea-time. **Other results:** Job coherence, job strain, and job satisfaction: all no change.
Berry et al. (2016)	UK 10 inpatient wards Mixed gender	Staff, patients *N* = 63 intervention arm (34.9% patients)	**Ward-based psychological intervention** Shared clinical formulation development for individual patients’ needs. Identification of specific patients’ strengths and “problem behaviors” based on hypotheses about links to and triggers of individual psychological distress. Discussion of implications for support plans.	Empirical link between staff-patient relationships and patient outcomes (e.g., Berry et al., 2011)	**Type:** Person-oriented **Complexity**: Somewhat	**Implementation:** 24 × 1-h sessions per ward over 6-month Delivered by Author 1: experienced psychologist **Fidelity arrangements:** No information re: fidelity	**Design:** Single-blind cluster randomized design (follow-up 6-month) **Climate measure:** WAS 22-item (Moos, 1974) **Results:** WAS: positive patient-reported changes in “system maintenance” and “relationships” dimensions **Other results:** Patient-reported perceived criticism by staff improved. Staff reported improved de-personalization (MBI)
Bjorkdahl et al. (2013)	Sweden 19 psychiatric wards (Emergency/admission, general, PICU, forensic)	Staff, patients *N* = 1567 (28.9% patients)	**Bergen violence prevention and management training programme** Aggression theory, ethics in care, ward rules and routines, risk factors and risk assessment, laws and legislations, impact of the physical environment. Limit-setting styles and negotiation, self-defense, physical & mechanical restraint, safety issues, seclusion and forced medication, post-incident debrief sessions with the patient, and critical review of violent incidents	City Model (Bowers, 2002); Public health: prevention model (International Labor Office, 2002)	**Type:** Person-oriented **Complexity:** Somewhat	**Implementation**: 4-day course 50% theory, 50% practical. Refresher classes at least 6-monthly based on current experiences Trainers from current active staff receive 70-h train the trainer course **Fidelity arrangements**: No information re: fidelity	**Design:** Pretest–posttest (follow up 6–9-month) **Climate measure:** E13 (Bjorkdahl et al., 2013) **Results:** Item-by-item analysis. Four E13 items for staff and one for patients improved
Bowers et al. (2015)	UK 31 acute mental health wards (16 intervention, 15 control)	Nursing staff (*N* = 564)	**Safewards interventions** (see Baumgardt et al., 2019 above)	See Baumgardt et al. (2019)	**Type:** Process/ Person-oriented **Complexity:** Definite	**Implementation**: Eight weeks to implement intervention and then continued use for a further 8 weeks Ward staff supported by 2–3 × weekly researcher visits **Fidelity arrangements:**: Post study self-report staff questionnaires (M = 89%)	**Design:** Blinded cluster RCT (follow-up 16-week) **Climate measure:** WAS (OO PC SC subscales only) (Moos, 1974) **Results**: No significant changes **Other results:** Conflict and containment (primary outcome) significantly reduced; self-harm and personality disorder-related attitudes, both unchanged; physical health improved in controls
Corey et al. (1986)	US Three mental health units (Acute, research alcohol) 102 beds in total	Staff, patients *N* = 247 (47.0% patients)	**Psychiatric ward refurbishing:** Changes in: furniture style, furniture arrangement, floor covering, color, ornamentation, individualization of living space. Authors note that conduct of pretest surveys themselves generated discussion and were a treatment component though this was not a priori a formal intervention. Target: patients and clinical staff.	Social ecology theory. (Proshansky et al., 1976)	**Type:** Structure, Process **Complexity:** Somewhat	**Implementation**: Refurbishment over 12-month period Consultants with experience of color and design with senior ward staff **Fidelity arrangements:**: No information	**Design:** Pretest–posttest (follow-up 14-month) **Climate measure:** WAS-R **Results:** Improvements for acute ward staff on Involvement (Inv), Aut; for research ward staff on PP; for acute psychiatry open patients on Inv, Aut, SC; for research ward patients on Inv, PPO; for alcohol patients on PPO
Eliassen et al. (2016)	Norway 2 × 10-bed units each with 30 staff. Mixed patients gender and diagnoses	Staff (*N* = 50; MBSR *n* = 27; AC *n* = 23)	**Mindfulness-Based Stress Reduction (MBSR) versus Affect Consciousness (AC):** MBSR: In group meetings, participants practiced mindful sitting and walking meditation, focus on bodily sensations, yoga exercises. Openness, friendliness, patience, and acceptance encouraged. Homework given. AC: Presentation and training about and expression of 11 basic emotions. The group was divided into subgroups of 3–4 people where they role played patient–therapist situation with the therapist conducting the AC interview. No homework.	Mindfulness. Staff members clarity about their own limits support psychological growth in patients	**Type:** Person-oriented **Complexity:** Somewhat	**Implementation**: Mindfulness: 8 × 90-min weekly group meetings + 30-min formal exercises per day, workbook, and CD for home practice. AC: similar group contact with training instructor. MBSR – Author 1 AC – Qualified psychologist **Fidelity arrangements:**: No information	**Design:** pretest–posttest (follow-up multiple to 12-month) **Climate measure:** WAS revised 80-item version (Rossberg & Friis, 2003a, 2003b) **Results:** Both interventions had positive short-term impact MBSR on Spt, AA, PC; AC on Spt, OO, PC, ES: **Other results:** Mindfulness: very limited improvement; General Milieu Index: some improvement
Frölich et al. (2018)	Switzerland 22 psychiatric wards	Staff (*N* = 91)	**Anti-aggression and de-escalation (ADE) training.** German language content.	Empirical link between ADE training and ward climate	**Type:** Person-oriented **Complexity:** Somewhat	5-day training in groups of 12–15 in 2015. Climate data collected 2016 and compared with extant 2012 data. **Fidelity arrangements**: No information	**Design:** pretest–posttest (pretest is extant data) **Climate measure:** EssenCES (Schalast et al., 2008) **Results**: improvement on PC and SS subscales plus on total score.
Gartshore (2018)	UK 20-bed acute mental health ward.	Staff, patients *N* = 28 (50.0% patients)	**Experienced Based Co-Design:** Liaise with senior staff, observation of environment, staff interviews, staff event, gather patient and carer experiences through filmed interviews, joint event, co-design phase, celebration and review event	Six-stage experience-based codesign quality improvement process (Robert et al., 2015).	**Type:** Process/ Person-oriented **Complexity:** Somewhat	Preexisting user group (“Research Net”) leads 6-stage process involving consultation/liaison with service users and staff. One-year project period. **Fidelity arrangements** King's Fund EBCD toolkit adherence e.g., time spent in joint staff/service user working	**Design:** pretest–posttest (follow-up 19-month) **Climate measure:** WAS-R **Results:** Patients: significant improvement on all subscales except PPO. Staff: no improvements on any subscale.
Gebhardt & Steinert (1999)	Germany. Four acute wards.	Staff, patients *N* = 345 ((53.0% patients)	**“Internal sectorization” of wards:** Operational reorganization from ward of admission based on gender/ clinical need to geographic catchment area. All wards moving towards sex integration and partial open-door policy. Same staff: patient ratio, number of beds, and treatment orientations otherwise.	Empirical research re: ward gender mix, open doors, and ward climate. Recognizes debate is essentially ideological	**Type:** Process **Complexity:** Low	Change to geographical sectorization. Follow-up surveys at 9-month and 18-month **Fidelity arrangements**: No information	**Design:** pretest–posttest (follow-up 9 and 18-month) **Climate measure:** WAS-R (German-language version) **Results**: Overall improvements in Aut, AA, OO **Other results:** Sexual aggression: slight increase Severe aggression: reduction
Haller et al. (1996)	US 16-bed locked inpatient unit	Staff *N* = 120	**Smoking ban:** No access to a smoking area on or off unit and no leave given to patients to be allowed to smoke.	Addictive psychopharmacological aspects of smoking	**Type:** Process **Complexity:** Low	1-month implementation period **Fidelity arrangements**: No information	**Design:** pretest–posttest (follow-up 2-month) **Climate measure:** WAS-S 40-item **Results:** No change **Other results:** Aggression, seclusion: no change
Hansen & Slevin (1996)	US Two (1 inter-vention, 1 control) wards in a psychiatric hospital.	Patients *N* = 36	**Introduction of therapeutic community principles** to a “pharmacological, custodial-oriented” ward: (1). Democratization of treatment planning by inviting patient; (2). Expressive rather than task-oriented approach to group therapy. Groups involved all patients and encouraged expression; (3) Modified program so all therapeutic activities occurred on the unit; (4) 2x daily community meetings where staff were to accept “bizarre” or “acting out” behavior	Group therapy is an important part of therapeutic community environment.	**Type:** Process/ Person-oriented **Complexity:** Somewhat	Prior to change treatment planning meetings held to formulate a plan and complete paperwork. **Fidelity arrangements**: No information	**Design:** pretest–posttest controlled (follow-up 1- and 2-month) **Climate measure:** WAS-R **Results:** Improvements for program unit on Inv, Spt, PO, PP relative to comparison unit by final follow-up).
Kerfoot et al. (2012)	UK. Three acute wards and one PICU (78 beds)	Patients *N* = 125	**Introduction of a dedicated psychology team providing “stepped care”.** The team offer: Ward-based daily groups, psycho-education, CBT, motivational interviewing techniques. Recovery-focused joint working with nursing staff. Direct work with patients: assessment, formulation, brief interventions. Consultation and formulation to inform care plans, crisis and discharge planning, run staff training events, policy development.	Not explicit	**Type:** Process/Person –oriented **Complexity:** Definite	Establishment of psychology team comprising: one part-time clinical lead psychologist, one full-time clinical psychologist, three assistant psychologists **Fidelity arrangements**: No information	**Design:** pretest–posttest (follow-up 24-month) **Climate measure**: EssenCES **Results:** Higher scores on all three EssenCES subscales (no inferential testing) **Other results:** Patients: Satisfaction with groups; treatment alliance, Length of stay; Readmission Staff: Satisfaction with psychology team; Recovery self-assessment provider version All positive but nonsignificant trends
Kristensen et al. (2015)	Denmark 10-outpatient and 9-inpatient units of a large psychiatric hospital	Staff *N* = 683	**Multicomponent leadership program** (1) leadership as a subject, (2) situational leadership and coaching, (3) managing communication, conflicts, and change, (4) motivation, development, and improvement (5) leading groups and teams. Individual supervision (up to 3 h per leader) by external psychologist	Empirical link between patient safety culture and care quality (Ostroff et al., 2013)	**Type:** Person-oriented **Complexity:** Definite	6-month implementation period 5 modules total 9 days Delivered by external industrial organizational psychologist **Fidelity arrangements**: No information	**Design:** pretest–posttest (follow-up 6-month) **Climate measure:** SAQ-DK (Kristensen et al., 2015) **Results:** Frontline staff positive changes: “teamwork climate”, “safety climate”, “job satisfaction”, “working conditions”, and “perception of management”. Leaders, improvement only on “stress recognition”.
Mistral et al. (2002)	UK One psychiatric ward (14-beds)	Health care staff *N* = 44	**Therapeutic community principles:** daily community meetings, mandated regular staff–patient care plan-focused interaction. Additional meetings (ward management focused on practical problems; monthly ward/community staff meeting; weekly supervision group). Clarification of aims and structure: clear rules and sanctions re: smoking, alcohol **Improved environment**: upgrade ward kitchens, bathrooms, carpets, paint **Improved safety:** staff alarms, training is risk assessment and control & restraint, police liaison developed.	Therapeutic community principles: belief in the importance of good communication between patients and staff, good ward environment, safety, clarity of aims and structures.	**Type:** Process/Person-oriented/Structure **Complexity:** Definite	Changes made over 6-month Ward manager responsible for implementation **Fidelity arrangements**: No information	**Design:** pretest–posttest (follow-up 6-month) **Climate measure:** WAS-R **Results:** Significant changes in Inv, PO **Other results:** Adapted attitudes scale: Improvements on skill & knowledge adequacy and self-esteem in this work. Reductions in seclusion and staff sick time over 2-year periods.
Ng et al. (1982)	Hong Kong 15-bed open ward and 12 patient day depart-ment	Patients *N* = 162	**Community meetings** (A). No meeting vs. (B). Medic-directed meeting focused on medical and ward management vs. (C). Nurse-directed meeting – nurses took to present for discussion individual patients vs (D). Nondirected unstructured meeting	Empirical links between meetings and ward incidents	**Type:** Process **Complexity:** Low	Implementation of 4 × 10-w blocks of no meeting [A], and 3x weekly medic-directed [B], nurse-directed [C], and nondirected [D] community meetings **Fidelity arrangements**: No information	**Design:** Longitudinal ABACADA (follow-up at 10-w, 20-w, 30-w) **Climate measure:** WAS-S **Results:** No significant difference **Other results:** Incidents: No significant differences
Nicholls et al. (2015)	Australia. One psychiatric unit.	Staff, patients *N* = 192 (52.1% patients)	**Relocation** to a new, custom-built acute mental health facility: Individual bedrooms, “socialization spine”, recreational courtyards, therapeutic spaces, family-friendly visiting space, prayer/reflection space	Environment/landscape and mental well-being – health geography (Philo, 1997)	**Type:** Structure **Complexity:** Low	9-mo implementation period Unclear responsibilities **Fidelity arrangements**: No information	**Design:** pretest–posttest (follow-up 9-month) **Climate measure:** WAS-R for staff, WAS-S for patients **Results**: Improvements for Staff *and* patients: OO, PC, and for staff on Inv
Pierce et al. (1972)	US One psycho-dynamically-oriented psychiatric ward in a general hospital..	Staff, patients *N* = 110 (19.1% patients)	**Ward Atmosphere Scale survey**. and discussion with ward staff about WAS results of staff and patients with resulting changes: structured community meeting; expanded activities program, limit-setting, improved communication of decisions, more private therapist use.	Psycho-dynamic: interplay of ego functions and external reality with ward social structure (Erikson).	**Type:** Process/ Person-oriented **Complexity:** Somewhat	2-month period of discussion of results and implementation period Responsibility for implementation with research team/ward staff **Fidelity arrangements**: No information	**Design:** pretest [A]–posttest (follow-up 5-month [B]and 7-month [C]) **Climate measure:** WAS 130-item (Moos, 1969) **Results:** Staff A to B six scales moved away from “Ideal” scores. B to C 9 moved towards ideal score, 2 away and 1 unchanged.
Rigby et al. (2001)	UK. Single acute psychiatric ward	Nursing staff *N* = 36	**Change from a keyworker to primary nursing system:** Literature distributed offering information on the new structure and clarifying new responsibilities, thus ensuring continuous care.	Primary nursing (Hall, 1969)	**Type:** Process, person-oriented **Complexity:** Low	Implementation of primary nursing over 4-month. Responsibility for implementation unclear **Fidelity arrangements**: No information	**Design:** pretest–posttest (follow-up 4-month) **Climate measure:** WAS-R **Results:** No significant improvements **Other results:** Staff questionnaire: general reduction in satisfaction
Southard et al. (2012)	US. One 50-bed unit in a psychiatric hospital.	Patients, staff *N* = 106 (76.4% patients)	**Change to an open nursing station from a “glass box”.** Baseline: nursing station was entirely enclosed by anti-shatter tempered glass. Follow-up: Glass removed, new furniture, paint, relocation of administrative staff from front to rear.	Closed nursing station demeaning (Henderson et al., 2007)	**Type:** Structure, Process **Complexity:** Low	24-month between pre- and post-measures. Unclear precisely when build occurred in that period. **Fidelity arrangements**: No information	**Design:** pretest–posttest (follow-up 24-month) **Climate measure:** WAS-R **Results:** No significant change for either group (any subscale)
Thorward & Birnbaum (1989)	Canada. One 17-bed locked acute care inpatient unit	Patients *N* = 152	**Smoking ban:** Baseline: smoking allowed in unit smoking room. Intervention: no smoking on unit or with staff during activities even outside the hospital. Patients could smoke outside on their own if they had sufficient outdoor “privileges”. NRT readily available.	Addictive psychopharmacological aspects of smoking	**Type:** Process **Complexity:** Low	2-month implementation before post-test measurement Unclear responsibility for intervention **Fidelity arrangements**: No information	**Design:** pretest–posttest (follow-up 2-month) **Climate measure:** WAS-R **Results**: positive changes: SC (all participants); OO (Patients); negative changes: OO (staff)
Urbanoski et al. (2013)	Canada. One 24-bed unit in a large hospital	Patients *N* = 290	**Ward relocation to 24-bed new build unit:** design is client-centered: private rooms with en-suite bathrooms, desk, and telephone; central common rooms, private visitation rooms, kitchen. Physical features and clinical routines changed simultaneously (not detailed)	Empirical link between ward climate and outcomes (Moos, 1986)	**Type:** Structure **Complexity:** Low	1-year in both existing and new locations: change implemented over 1-year. Unclear responsibility for delivery **Fidelity arrangements**: No information	**Design:** pretest–posttest **Climate measure:** WAS (Spt, PO, Aut only) **Results**: Significant improvements on all subscales **Other results: Improvements on** quality of life, functioning and treatment satisfaction.

### Data Synthesis

**Figure 1.**
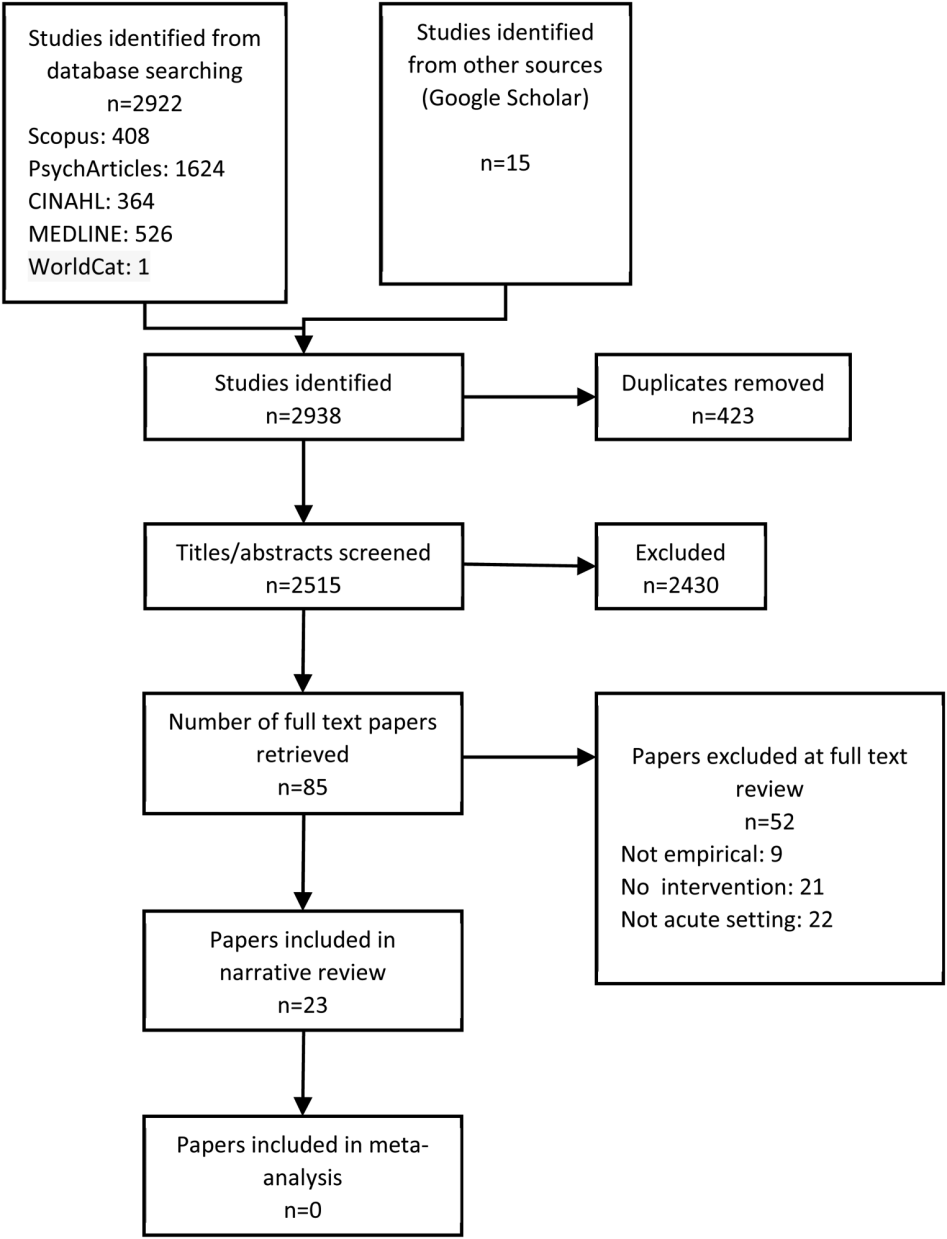
PRISMA flow diagram of literature search strategy.

Quantitative results regarding climate change associated with an intervention were extracted (see Table 4) and, where possible, the standardized mean effect size (Cohen's *d*) and 95% CI calculated. Where studies presented information separately for different, nonoverlapping samples (e.g., patients and staff) we treated them individually. Where studies reported on multiple follow-up points we used that from the longest follow-up period reported. Meta-analyses were not conducted due to insufficient between-group trials. Standardized mean differences from pretest–posttest studies cannot distinguish between intervention and contextual (e.g., natural progression, follow-up time variation) effects (Cuijpers et al., 2017). Where more than one study contributed data for the same outcome (i.e., subscale or total scale score), effect sizes were plotted (see Figure 1). For the E13 (Bjorkdahl et al., 2013), where results from the single study using this measure were reported on an item-by-item basis, the mean E13 effect size was calculated as a summary indicator. All other information was subject to descriptive statistics and narrative synthesis.

**Table 4.** Study Effect Sizes (*d*) for Staff and Patient Samples from Evaluation Studies Using the Ward Atmosphere Scale.

		Ward Atmosphere Scale subscale												
Study	Group measured	Involvement	Support	Spontaneity	Autonomy	Practical Orientation	Personal Problem Orientation	Anger & Aggression	Order & Organization	Programme Clarity	Staff Control	System Maintenance	Relationships	Personal Growth
Aubry et al. (1996)	Staff	0.06 (−0.66, 0.77)	−0.49 (−1.2, 0.25)	0.46 (−0.28, 1.17)	0.59 (−0.16, 1.30)	0.12 (−0.60, 0.84)	0.44 (−0.30, 1.15)	0.53 (−0.21, 1.24)	−0.66 (−1.37, 0.09)	−0.19 (−0.91, 0.53)	1.31 (−0.05, 2.06)	−	−	−
Southard et al. (2012)		−0.14 (−0.92, 0.65)	0.52 (−0.29, 1.30)	−0.22 (−1.0, 0.57)	−0.39 (−1.17, 0.41)	0.35 (−0.46. 1.12)	0.02 (−0.77, 0.8)	−0.07 (−0.71, 0.86)	0.11 (−0.68, 0.89)	0.12 (−0.67, 0.9)	−0.70 (−1.48, 0.13)	−	−	−
Nicholls et al. (2015)		0.38 (−0.03, 0.79)	0.22 (−0.19, 0.63)	0.09 (−0.32, 0.50)	−0.08 (−0.49, 0.33)	0.00 (−0.41, 0.40)	**0.45** (0.04, 0.86)	**0.42** (0.0, 0.83)	**0.58** (0.16, 0.99)	**0.52** (0.1, 0.94)	0.16 (−0.25, 0.57)	−	−	−
Eliassen et al. (2016)a		0.32 (−0.22, 0.86)	**0.69** (0.13, 1.23)	−	−	0.23 (−0.31, 0.76)	−	**0.62** (0.06, 1.15)	0.42 (−0.13, 0.95)	**0.75** (0.18, 1.29)	0.08 (−0.45, 0.61)	−	−	−
Eliassen et al. (2016)b		−0.28 (−0.85, 0.31)	−0.35 (−0.93, 0.23)	−	−	−0.28 (−0.85, 0.31)	−	0.26 (−0.33, 0.83)	0.12 (−0.46, 0.70)	−0.25 (−0.83, 0.33)	−0.10 (−0.68, 0.48)	−	−	−
Mistral et al. (2002)		0.26 (−0.34, 0.85)	−	−	−	**0.90** (0.27, 1.5)	−	−	−	−	−	−	−	−
Gartshore (unpublished)		0.35 (−0.41, 1.09)	0.59 (−0.18, 1.33)	0.42 (−0.34, 1.16)	0.43 (−0.33, 1.17)	0.40 (−0.36, 1.13)	0.07 (−0.67, 0.81)	0.63 (−0.15, 1.37)	0.37 (−0.39, 1.10)	0.52 (−0.25, 1.26)	0.32 (−0.43, 1.06)	−	−	−
Berry et al. (2016)		−	−	−	−	−	−	−	−	−	−	0.08 (−0.35, 0.52)	0.29 (−0.15, 0.72)	0.20 (−0.24, 0.63)
Urbanoski et al. (2013)	Patients	−	**0.36** (0.13, 0.59)	−	**0.79** (0.55, 1.03)	0.19 (−0.04, 0.42)	−	−	−	−	−	−	−	−
Aubry et al. (1996)		0.06 (−0.45, 0.57)	**0.91** (0.36, 1.43)	0.46 (−0.06, 0.97)	**0.87** (0.33, 1.39)	**0.66** (0.13, 1.18)	0.39 (−0.13, 0.9)	**0.83** (0.29, 1.35)	**0.71** (0.18, 1.23)	**0.97** (0.42, 1.50)	**0.57** (0.05, 1.09)	−	−	−
Southard et al. (2012)		−0.18 (−0.62, 0.25)	−0.17 (−0.61, 0.26)	0.11 (−0.32, 0.55)	0.05 (−0.39, 0.48)	0.31 (−0.13, 0.74)	−0.05 (−0.49, 0.38)	−0.12 (−0.55, 0.38)	−0.13 (−0.56, 0.31)	−0.06 (−0.49, 0.38)	−0.14 (−.58, 0.30)	−	−	−
Nicholls et al. (2015)		**0.50** (0.1, 0.89)	0.24 (−0.16, 0.63)	**0.45** (0.05, 0.64)	**0.43** (0.03, 0.82)	**0.49** (0.8, 0.88)	0.11 (−0.29, 0.50)	0.11 (−0.28, 0.5)	**0.88** (0.46, 1.28)	**0.40** (0.0, 0.79)	**0.65** (0.25, 1.05)	−	−	−
Hansen & Slevin (1996)		−0.33 (−1.1, 0.47)	**0.94** (0.10, 1.73)	−0.09 (−0.86, 0.70)	0.41 (−0.39, 1.19)	0.40 (−0.4, 1.18)	0.02 (−0.76, 0.8)	−0.19 (−0.97, 0.60)	−0.17 (−0.95, 0.61)	0.19 (−0.59, 0.97)	0.11 (−0.67, 0.89)	−	−	−
Gartshore (unpublished)		**1.06** (0.24, 1.82)	**1.09** (0.27, 1.85)	**0.74** (0.04, 1.59)	**0.80** (0.01, 1.55).	**1.07** (0.25, 1.82)	0.01 (−0.74, 0.75)	0.52 (−0.25, 1.26)	**1.18** (0.35, 1.98)	**1.21** (0.37, 1.98)	**1.11** (0.28, 1.17)	−	−	−
Berry et al. (2016)		−	−	−	−	−	−	−	−	−	−	**1.18** (0.52, 1.79)	**3.23** (2.28, 4.06)	**0.8** (0.23, 1.47)

a Mindfulness sample; b Affect consciousness sample; negative sign (−) means negative outcome for the subscale (e.g., for “staff control”—signifies an increase in the amount of control staff use). The scales reported by Berry et al. (2016) are composites of subgroups of WAS subscales. **Bold** indicates significance based on 95% CIs.

### Most Promising Interventions

In order to summarize the extent to which study interventions have been successful, we tracked each study against the following criteria.

1. Intervention inclusiveness: the intervention targeted current inpatients unless it was otherwise explicit that they were intended to be *indirect* beneficiaries of an intervention targeted at staff.
2. Evaluation inclusiveness: the social climate-related outcome for the intervention included inpatient ratings.
3. Study quality: assessed quality to be at least moderate *and* the theoretical basis of the intervention-outcome link should be explicit and plausible.
4. Positive findings: climate-related findings should be positive for inpatients and not detrimental for staff.
5. Findings triangulated: study includes additional nonsocial climate-related outcomes which corroborate social climate findings.

## Results

The search strategy resulted in the inclusion of 23 studies published between 1972 and 2019. Almost half (10/23, 43.5%) were published since 2013. Studies were conducted in ten countries (US *n* = 6; UK *n* = 6; Canada *n* *=* 3; Germany *n* = 2; Australia, Denmark, Hong Kong, Norway, Sweden, and Switzerland all *n* = 1). Twelve were conducted on a single ward and eleven (47.8%) across multiple wards or sites (range 2–22, median = 3). Eight studies were published in nursing journals (*n* *=* 7 specialist mental health nursing journals), *n* = 8 in medical journals (*n* = 7 specialist psychiatry journals), *n* = 4 service-focused journals, *n* = 2 psychology journals; and *n* = 1 study was an unpublished PhD thesis.

### Study Quality

Thirteen (56.5%) studies received a global quality rating of weak and the remaining ten were rated moderate. Common sources of bias were lack of blinding, high withdrawal rates, and lack of clarity around study confounders (see Table S1). Of the more complex interventions (see below), studies rated as moderate in quality were those of Berg and Hallberg (1999), Berry et al. (2016), Eliassen et al. (2016), Kristensen et al. (2015), Bowers et al. (2015), Baumgardt et al. (2019), and Mistral et al. (2002).

### Study Design

Twenty (87.0%) studies used an uncontrolled pretest–posttest design. Of the remainder, Berry et al. (2016) used a single-blind cluster randomized design in which staff and patients on ten wards were assigned to treatment as usual or to a program of 24 × 1-h weekly patient-focused group supervision sessions. Bowers et al.’s (2015) Safewards cluster RCT allocated 31 wards to either a theoretically grounded conflict reduction model or a generic well-being model. Finally, Hansen and Slevin (1996) compared nonrandomly assigned intervention (*n* = 2) and nonintervention wards (*n* = 2).

### Study Interventions

Study interventions were heterogeneous in type and complexity (see Table 3). Twelve (52.2%) comprised a single intervention type. Two interventions were solely environmental/structural in nature, both describing interventions involving relocation to new clinical premises (Nicholls et al., 2015; Urbanoski et al., 2013), and both were judged low in complexity. Environmental/structural changes were also present alongside other intervention types in four further studies. Southard et al. (2012) described improving accessibility to a central nursing station via removal of the goldfish bowl style glass, while Corey et al. (1986) and Mistral et al. (2002) described extensive ward redecoration and refurbishment, and Aubry et al. (1996) described ward relocation. Three study interventions were categorized as person-focused in entirety, two being violence prevention, management and de-escalation training packages (Bjorkdahl et al., 2013; Frölich et al., 2018) and one focused on improving clinicians’ emotional regulation through mindfulness-based stress reduction or affect consciousness (Eliassen et al., 2016). These three interventions were all judged to be somewhat complex. Five studies tested interventions that solely comprised process elements and all five were judged to be low in complexity: Haller et al. (1996) and Thorward and Birnbaum (1989) both described smoking bans, Ng et al. (1982) trialed community meetings led by medics, nurses, or neither. Rigby et al. (2001) described a literature-supported shift from keyworker to primary nursing, and Gebhardt and Steinert (1999) presented findings from an evaluation of “internal re-sectorization” involving a behind-the-scenes procedural shift from admission to a mental health ward based on clinical acuity and gender to one based on one's home geographical location.

Chronologically, studies published pre-1990, with the exception of Pierce et al. (1972), described low complexity interventions; over time, studies increased in frequency *and* complexity. The more complex interventions included the introduction of the Safewards conflict and containment reduction program (Baumgardt et al., 2019; Bowers et al., 2015); programs based on principles of therapeutic community-style approaches (Hansen & Slevin, 1996; Mistral et al., 2002); supervised psychological (Berry et al., 2016) or nursing (Berg & Hallberg, 1999) supervision; a new clinical psychology team (Kerfoot et al., 2012); a leadership program (Kristensen et al., 2015); a mindfulness program (Eliassen et al., 2016); and a Ward Atmosphere Scale-derived needs assessment with associated action planning (Aubry et al., 1996).

Most papers offered some level of theory-based rationale underlying the choice of the study intervention. At the least well-articulated level, study authors simply noted a lack of evidence for current practice and a corresponding desire to solve a local issue such as how to facilitate community meetings (Ng et al., 1982). Others also did not refer to formal theory but noted well-established empirical links between aspects of the ward social climate and relevant outcomes (e.g., Aubry et al., 1996; Berry et al., 2016). At the most well-articulated level, interventions such as Safewards (Bowers et al., 2015) explained and made reference to well-drawn theory involving both plausible mechanisms of action for the interventions’ components and literature-based and empirically derived supporting evidence.

Across studies, interventions were delivered solely to clinical staff, solely to patients, and to both. When an intervention was delivered to staff there was an explicit or implicit intention of indirect patient benefit; for example, interventions such as mindfulness-based stress reduction (Eliassen et al., 2016) or aggression management training (Frölich et al., 2018) were delivered solely to staff but an anticipated outcome was improved social climate. The Safewards conflict reduction intervention (Bowers et al., 2015; Baumgardt et al., 2019) involves elements that are aimed clearly at staff (e.g., “say something positive about each patient in nursing handover”) and at patients (e.g., “staff to offer reassurance to patients in disturbed ward situations”). Interventions comprising a structural change such as ward-relocation, or an extensive operational/process-type intervention could scarcely be said to apply only to either patients or staff. In these instances, however, we distinguished the target group based on whether solely staff, patient, or both perspectives on ward climate change were sought in the intervention evaluation; for example, Hansen and Slevin (1996) and Urbanoski et al. (2013) reported on extensive operational and structural interventions but drew conclusions about climate change solely from patient-rated climate measures. Based on these criteria, twelve studies offered interventions directly to both staff and patients, six directly to staff only, and five to patients only. Of interventions aimed at patients only, two have just been described (Hansen & Slevin, 1996; Urbanoski et al., 2013), and two comprised imposed smoking bans with little in the way of additional support (Thorward & Birnbaum, 1989; Haller et al., 1996), and one a relatively simple manipulation of community meeting facilitation (Ng et al., 1982).

### Study Participants

Study participants comprised a group or groups overlapping with those who had been targeted by the study intervention. For example, smoking bans appeared to be directed solely at patients (Haller et al., 1996; Thorward & Birnbaum, 1989) but the study participants for the former study were staff-only while those for the latter were, more congruently, patients-only. Kerfoot et al.’s (2012) description of the establishment of a new psychology team with clear staff- *and* patient-directed intervention elements used the EssenCES outcome scale only with the patient group. In summary, participants in studies were ward staff (*n* = 18) and patients (*n* = 15); nine studies recruited both and in each case results were reported separately. The total number of participants was 3,475 staff (2,118 baseline and 1,445 follow-up) and 1,810 patients (980 baseline and 910 follow-up). Most (19/23; 82.6%) studies had a single follow-up point; Gebhardt and Steinert (1999), Hansen and Slevin (1996), and Pierce et al. (1972) all made the third measurement; Ng et al. (1982) followed-up after each of three variations of the intervention used which comprised different approaches to facilitating ward community meetings; and Eliassen et al. (2016) measured on six occasions from pre-baseline to 12 months. In pretest–posttest studies, the second cohort was, due to patient discharge or staff turnover, rarely the same individuals as the first. Only Kristensen et al. (2016) explicitly collected sufficient details to ensure that follow-up data could be matched at an individual level.

### Climate-Related Outcomes Measures Used

Five different climate-related scales were used across the included studies:

#### Ward Atmosphere Scale

The Ward Atmosphere Scale (Moos, 1986) was the most commonly used measure (*n* = 17; 77.3%). The tool's manual states it is “suitable for impact evaluation of intervention programs” (Moos, 1989). Several variations of the tool were used, most commonly (*n* = 12 studies) the 100-item “real” scale (Moos, 1989) comprising 10 subscales theoretically grouped into three “higher order” dimensions (Dimension 1: Relationships [“involvement”, “spontaneity”, “support”]; Dimension 2: Personal growth [“autonomy”, “practical orientation”, “personal problem orientation”, “anger and aggression”]; Dimension 3: System maintenance [“order and organisation”], “programme clarity”, “staff control”). In one instance where the “real” scale was used solely with staff (Nicholls et al., 2015), a sample of patients completed a 40-item “short” (Moos, 1989) version of the tool. The “short” tool replicates the first 40 items of the “real” version and is reportedly interchangeable with it. The 40-item tool was itself used in one other study with a staff-only sample (Haller et al., 1996). Other studies using the Ward Atmosphere Scale involved a 22-item version (Moos, 1974) comprising three subscales mirroring the three higher order domains of the 100-item version (Berry et al., 2016), an 80-item 11-subscale amended version (Rossberg & Friis, 2003a, 2003b) used in Eliassen et al., 2016), a 130-item 12 subscale version (Moos, 1969 used in Pierce et al., 1972), a 30-item version comprising the “spontaneity”, “autonomy”, and “problem orientation” “real” subscales only (Moos, 1986, used in Urbanoski et al., 2013). All the variations of the tool described here comprised statements to which respondents are required to respond “True” or “False”. No included study provided information about the factor structure of the tool based on their own data; only Nicholls et al. (2015) provided information about the internal consistency of the data for their own sample noting that scales relating to “spontaneity”, “autonomy”, “anger and aggression”, and “personal problem orientation” were dropped from analyses due to unacceptable Cronbach's alphas. A number of studies referenced prior studies which they claimed supported the case for the reliability and convergent/divergent validity of the tool.

EssenCES (Schalast et al., 2008) was used in three studies. EssenCES is a 15-item three-scale (“therapeutic hold”: the extent to which the ward is perceived as supportive of patients’ therapeutic need; “experienced safety”: the extent to which staff and patients feel safe on the ward; and “patients’ cohesion”: the extent to which patients care for and support one another) tool for patients and staff. Response is on a five-point Likert scale (0 = not at all; 1 = little; 2 = somewhat; 3 = quite a lot; 4 = very much). The tool was developed and validated in a German-language version but has been translated and validated in an English-language version subsequently. No included study (Baumgardt et al., 2019; Frölich et al., 2018; Kerfoot et al., 2012) provided data about the EssenCES internal consistency or factor structure specific to the study sample, but its psychometric properties are well-documented (e.g., Tonkin et al., 2012; Tonkin, 2016) at least for forensic settings. It has not been validated for use in civil/non-forensic settings.

#### E13

The E13, described by its author as a scale to measure violence prevention and management climate on inpatient units (Bjorkdahl et al., 2013), comprises 13 items, response is on a four-point scale (1 = not at all; 2 = unspecified; 3 = unspecified; 4 = totally), which are dichotomized to “agree”/“disagree” for analyses. Factor analysis revealed a three-factor structure explaining 52.3% of variance; however, internal consistency of factors 2 and 3 was inadequate and the authors report a 1-factor solution (Cronbach's alpha = 0.83) to be preferable. However, study results were reported on an item-by-item basis rather than for the whole scale. No data about the convergent/divergent validity or the reliability (e.g., test–retest) were presented.

#### Safety Attitudes Questionnaire Danish version

Sexton et al. (2006) is a six-factor (“teamwork climate”, “safety climate”, “job satisfaction”, “stress recognition”, “working conditions”, and “perception of management”), 31-item tool which aims to capture quantitative measurements of patient safety culture. The single study (Kristensen et al., 2016) to use the tool included in this review reported acceptable Cronbach's alphas (>0.70) for scales other than “teamwork climate” and “safety climate”. The authors state that their own previous work (Kristensen et al., 2015) has established that the tool is “psychometrically sound”.

#### Creative Climate Questionnaire (CCQ)

The Creative Climate Questionnaire (Ekvall et al., 1983) is a 50-item tool covering 10 dimensions (challenge, freedom, idea-support, trust, dynamism, playfulness, debates, conflict, risk-taking, and idea-time). Items are rated 0 = absolutely inapplicable to 3 highly applicable with a high score representing a more creative climate. In this review, the CCQ was used solely by Berg and Hallberg (1999). No data on internal consistency for the study sample were presented; however, a number of significant correlations between CCQ dimensions and the Satisfaction with Nursing Care and Work (SNCW) scale (Hallberg et al., 1993) are reported.

### Study Findings

Social climate-related outcomes were gathered from staff and patients in 18 and 16 studies, respectively, including from both in 11 studies. Nonclimate-related outcomes were gathered in eight studies each for staff and patients including from both in four studies. Where sufficient information was presented to calculate the effect size for a climate-related outcome a total of 13 of the included studies involving 21 samples of either patients (*n* = 2), staff (*n* = 5), or both (*n* = 7) yielded 148 unique results from combinations of climate scale subscale score and intervention. The majority were (115/147; 77.7%) for the Ward Atmosphere Scale subscales. Effect sizes were not calculable from ten papers due to the lack of information. Individually, these studies reported positive changes as measured on the Ward Atmosphere Scale (Pierce et al., 1972) and EssenCES (Kerfoot et al., 2012), no significant change in the Ward Atmosphere Scale (Bowers et al., 2015; Haller et al., 1996; Ng et al., 1982; Rigby et al., 2001), mixed findings on the Ward Atmosphere Scale including little change over five months but more positive changes over six (Pierce et al., 1972), and—as expected given the nature of the intervention (smoking ban)—negative changes on the Ward Atmosphere Scale in terms of increased perception of staff control (Haller et al., 1996). Bjorkdahl et al.’s (2013)_ENREF_3 investigation of the violence prevention climate revealed significant change using the E13 individual item measures at six-month follow-up for staff on four items and for patients on one item.

#### Ward Atmosphere Scale intervention effect sizes: staff

Data extraction led to *n* = 59 unique sample-subscale combinations (see Table 4) from nine samples reported in eight studies (follow-up range 6–24 months, median = 12 months). There were eight unique subscale effect sizes with statistically significant change based on inspection of 95% confidence intervals. All involved change in the desired direction. Of these, effect sizes were small (<0.5), medium (0.5–0.74), and large (>0.75) for three, three, and two combinations, respectively. Significant effect sizes were spread over six subscales with only the anger and aggression and personal problem orientation subscales having significant effect sizes in two studies.

#### Ward Atmosphere Scale (Moos, 1986) intervention effect sizes: patients

Data extraction led to *n* = 56 unique sample-subscale combinations from seven studies (median follow-up period of 12 months, range 6–24 months). There were 27 statistically significant effect sizes of small (*n* = 5), moderate (*n* = 5), and large (*n* = 17) magnitude, all in the desired direction. All subscales were represented except personal problem orientation, and all but anger and aggression also had a significant effect size contributed from more than one study. All three composite scales reported by Berry et al. (2016) were significant and large in magnitude.

#### Other climate scales intervention effect sizes

Examination of studies using other climate scales yielded 25 effect sizes of which 21 involved staff. Of the studies using the EssenCES, Fröhlich et al.’s (2018) data revealed small and moderate effect sizes for staff-rated improvement on the “patient cohesion” (*d* = 0.47 [95% CI 0.05, 0.88]), and “subjective safety” (*d* = 0.51 [0.09, 0.92]) subscales, while Baumgardt et al.’s (2019) study revealed a small effect size for improvement in “therapeutic hold” for staff (*d* = 0.40 [0.0, 0.81]) and a moderate effect size for patients (*d* = 0.50 [0.05, 0.94]). Calculation of effect sizes from Berg and Hallberg’s (1999) study using the Creative Climate Questionnaire report revealed a significant change among nurse respondents only on the “idea-time” subscale (*d* = 0.73 [0.05, 1.40]). Finally, Kristensen et al.’s (2016) evaluation of a multicomponent leadership program at a six-month follow-up using the Safety Attitudes Questionnaire resulted in small positive effect sizes on the subscales related to safety climate (*d* = 0.22 [0.07, 0.37]) and job satisfaction (*d* = 0.35 [02, 0.5]).

#### Climate-related change: effect size by intervention type and complexity

For the Ward Atmosphere Scale, two studies, one evaluating the introduction of therapeutic community principles (Mistral et al., 2002), and one an entire new unit build (Nicholls et al., 2015), reported positive moderate or large effect sizes for two staff-reported subscales each and no equivalent negative changes. Berry et al.’s (2016) analyses of data from pre- and post-introduction of regular individual-patient focused formulation training for staff revealed large effect sizes for patient-reported but not staff-reported Ward Atmosphere Scale scores on all three composite variables. Significant improvements were recorded on four and seven Ward Atmosphere Scale subscales by patients in two studies (Aubry et al., 1996; Nicholls et al., 2015). Thus, results from two studies of the effect of environmental change, and one each of process or policy change, and staff education and support, indicated positive benefit and no negative effects. Eliassen et al.’s (2016) study revealed moderate to large effect sizes for three Ward Atmosphere Scale subscales in relation to the use of mindfulness-based stress reduction, and five for the affect consciousness intervention; however, the effects were negative in one and four cases respectively. From studies using other outcome measures, moderate effect sizes were reported using the EssenCES to evaluate interventions to reduce violence and other conflicts.

#### Non climate-related findings

More than half (*n* *=* 11) of the included studies reported data from a total of 27 additional nonclimate-oriented measures (see Table 4 for details). Of these, significant change following an intervention was detected on measures of patients’ perception of staff criticism and staff depersonalization (Hansen & Slevin, 1996), staff mindfulness (Eliassen et al., 2016), patient aggression (Hansen & Slevin, 1996), complaints, aggression and negativistic behaviors (Ng et al., 1982), ward incidents (Mistral et al., 2002), staff self-perceived skill and knowledge adequacy (Southard et al., 2012), incidents of patient conflict and staff containment behaviors, and physical health (Hansen & Slevin, 1996). The significant change was not detected on measures of sense of coherence, work-related strain, satisfaction with nursing care (Berg & Hallberg, 1999), psychopharmaceutical use (Thorward & Birnbaum, 1989), staff attitudes (Mistral et al., 2002), working alliance, staff perceived criticism, general health, schizophrenia symptoms, and general patient functioning (Berry et al., 2016).

### Identifying Promising Interventions

Application of criteria for assessing the potential value of study interventions are presented in Supplementary Material (Table S2). Interventions of clear value were described in Berry et al.’s (2016) account of psychological formulation for multiple outcomes, and Baumgardt et al.’s (2019) Safewards implementation study. Bowers et al. (2015) own Safewards intervention was clearly valuable in relation to their own selected primary outcome, conflict and containment, but it did not yield improvements in terms of ward climate. Nicholls et al.’s (2015) intervention value was only compromised by a lack of other measures to corroborate the positive change wrought by ward relocation. Studies of other interventions were compromised across more criteria. Thus, of 23 studies, the interventions of most promises in relation specifically to improving social climate were those described in the studies of Aubry et al. (1996), Baumgardt et al. (2019), Berry et al. (2016), and Urbanoski et al. (2013).

## Discussion

This review has identified and synthesized the existing empirical evidence relating to interventions for social climate improvement in acute inpatient mental health settings. It has focused on the effectiveness of interventions to improve outcomes on direct measures of climate rather than proxies. An array of relevant measures have been discussed in the literature, but a limited number have been used in inpatient mental health settings; fewer still have been used to gauge the effectiveness of an intervention. We identified 23 relevant studies conducted in 10 countries and published over 47 years to 2019. Using heuristic criteria related to study quality, user involvement, and positive findings we identified only four studies yielding promising results. Of these, Baumgardt et al. (2019) described the Safewards conflict and containment reduction program, an intervention with a good evidence-base for effectiveness in terms of its intended target outcome (Bowers et al., 2015) but not primarily predicated on social climate change. Studies by Urbanoski et al. (2013) and Aubry et al. (1996) relied, respectively, wholly or partly on ward relocation. Opportunities to evaluate the effect of such changes are rare and should be grasped when they arise; however, they are likely always to be precisely opportunistic. Berry et al. (2016) successfully delivered an educational and developmental intervention whose target was the social climate and staff–patient relationships. Thus, one conclusion of the review must be that, in terms of planned interventions that are not reliant on capital spending, conflict reduction programs such as Safewards and educational and developmental initiatives such as Berry et al.’s (2016) use of clinical supervision and psychological formulation, are the most promising for improving measured climate.

Of the utilized measures, only Berg and Hallberg’s (1999) evaluation of the effect of group supervision used one that might be best defined as focusing on organizational culture (CCQ). Kristensen et al.’s (2016) study of a leadership safety program focused on safety climate, while Bjorkdahl et al. (2013) examined changes in the violence prevention climate. All other included studies focused on measures of the therapeutic climate, namely the Ward Atmosphere Scale or EssenCES. Thus, there is very little evidence about the effect of interventions on the *organizational culture, safety culture, or violence prevention climate* in these settings. Although the aim of the current study was not to conduct an exhaustive search of the effect of interventions for either organizational or therapeutic change as measured on *any* instrument, we are confident that our search strategy has detected the main scales used and the studies in which they have been used to gauge intervention effectiveness.

There is ample evidence that measures of therapeutic or other cultural climate types are associated with important patient-related therapeutic outcomes; however, our review has shown that the investigation of this and related constructs themselves, particularly their robustness and sensitivity to change over time, is demonstrably lacking. There are two potential explanations for this. First, it is possible that the interventions themselves are not effective at enhancing social climate. Alternately or additionally, the questionnaires used to measure social climate might not be measuring climate in a reliable or valid way, thus meaning that any changes that have occurred as a result of the interventions are simply not captured by existing questionnaires. In relation to this latter explanation, the EssenCES was used in several of the studies included in the current review. This is potentially problematic because the EssenCES was designed for use in forensic settings and has not been validated for use in non-forensic, acute mental health settings. It is possible that social climate differs fundamentally between these settings. If so, the EssenCES may not capture the relevant components of climate and would be unlikely to capture post-intervention changes in climate within non-forensic, acute mental health settings. Regarding the WAS (which was used in the majority of studies identified in the current review), there is not convincing evidence to support its psychometric properties (see Tonkin, 2016) and a number of further criticisms have been raised of the WAS, including outdated content and lengthy completion times which may be unsuitable for certain psychiatric populations (Schalast et al., 2008). One important direction for future research is, therefore, to identify measures of the social climate that are validated for use in non-forensic settings. If none exist, then future research should begin to explore whether existing measures (e.g., the WAS and EssenCES) can be reliably used to measure climate in non-forensic settings.

Associations between important outcomes and climate-related constructs are commonly used to justify clinical approaches or proselytize for new research. However, if those associations do not result in a change in the construct of interest when they are trialed we should treat such claims with appropriate skepticism. Of all the included studies, those of Berry et al. (2016), incorporating control and randomization elements, and Bowers et al.’s (2015) Safewards cluster randomized controlled trial were the most robust. The former reported some of the most convincing results of Ward Atmosphere Scale improvement for patients, though not for staff. While it is intriguing to consider why this might be the case it may not be too surprising that patients proved more sensitive to change in ward atmosphere than staff. Unfortunately, a lack of available follow-up data (Bowers et al., 2015) and non-equivalency of Ward Atmosphere Scale subscale reporting (Berry et al., 2016) somewhat limits the findings of these studies.

It is unsurprising that widely diverging intervention types have the potential to contribute to an improved climate. The three broad types identified in this review—environmental, operational, and developmental—though not necessarily the individual interventions within each type—have, at face value, plausible mechanisms of operation. That an optimum intervention might include capital spending on environmental improvement together with comprehensive staff development and education, and optimum, evidence-based practices and procedures seem axiomatic. However, and especially in the current economic climate, the opportunity to have the stars align so fortuitously makes the possibility of an opportunity to robustly evaluate the occurrence of such foresighted investment vanishingly small. In the event of any such initiative, it is crucial that lessons are learned about the shortcomings of study designs utilized thus far. The current study has demonstrated that there is a clear lack of robust evidence examining the impact of interventions designed to enhance social climate. As such, there is a need for more robust research designs in future. Specifically, research that utilizes a pretest–posttest design, multiple baseline measurements, multiple follow-ups, use of different and multiple scales is much-needed in order to fill the gap in high-quality evidence that is currently lacking. That more than a third of studies were published in nursing journals speaks to the importance of this topic of research for the nursing profession and reflects the importance nurses and nursing academics have in ensuring the developing evidence base reflects the nursing role in climate improvement.

From the current study, the only climate-related outcome measure which proved promising in terms of its sensitivity to change was the Ward Atmosphere Scale *practical orientation* subscale, and then this only achieved a small effect size across studies. Since the designs of included studies were limited, we were unable to conduct meta-analyses and, as a result, any significant moderation of results by intervention type or outcome measure remain uninvestigated. Thus, while it is tempting to hypothesize that different interventions might be linked to different potential outcomes, we cannot point to any positive evidence of this. Similarly, participant group (staff vs. patient) and study quality (low vs. moderate) are potential moderators.

The above creates something of a dilemma since, while the current evidence base does not support the routine use of the currently available measurements to evaluate the effectiveness of interventions to improve climate, it remains incumbent on services to attempt to justify such efforts. One solution might be to abandon climate-oriented measures and focus instead on proxy measures of improvement. However, even here the evidence is somewhat equivocal with nonclimate measures including satisfaction, quality of life, mindfulness, job strain, and aggression detecting change in about a little over half of the cases where they were used. To an extent, therefore, any attempts to develop better, more sensitive measures of climate and culture may be justified, but the assumption that the existing tools are not capturing actual change is not strongly warranted by the extant evidence. Certainly, further use of the EssenCES to determine whether it might be a more appropriate outcome measure is warranted since it has not had sufficient use to make firm conclusions about its sensitivity; however, it was not designed as a measure for non-forensic settings. Any development of new instrumentation should endeavor to maximize the ability to detect change as well as other more usually investigated psychometric properties; at the same time, tools need to be stable where this is warranted. Given the nonconclusive findings of climate and culture construct measures, it is prudent to incorporate other measures into evaluations when they are not already done so. However, these should be carefully selected and theoretically justified in relation to the study intervention. For example, Berry et al.’s (2016) study provides an exemplar of well-chosen supplementary measures of staff–patient relationships which are congruent with study aims.

### Study Limitations

The study has two main limitations. First, the overall quality of included studies was suboptimal, making generalizations from these results problematic. Second, meta-analysis was not possible due to problems with the study design. Although it certainly is possible to conduct meta-analyses on pretest–posttest studies, it is considered unwise due to the inability to disentangle intervention from time or miscellaneous effects and due to the large amounts of heterogeneity in study design, outcomes, follow-up periods, and so on (Cuijpers et al., 2017).

## Implications for Practice

The current review shows that, in the absence of significant new funding for wholesale environmental relocation of services, the interventions most likely to bring about change in social climate on acute mental health wards are the Safewards package of conflict and containment reduction measures (Bowers et al., 2015) and the psychological formulation approach described by Berry et al. (2016). Nurses should consider working towards the introduction of either or both of these approaches where it is appropriate.

## Conclusion

Efforts to evaluate the impact of interventions to improve the conditions which prevail in mental health units are important. This paper provides the first review of the evidence on this topic, finding that—despite at least 40 years of research—there is a surprisingly small amount of published evidence on the topic. Furthermore, the research that does exist is generally of poor methodological quality. For many reasons, social climate remains an important construct in acute mental health settings, both in terms of ensuring that patients are provided with the best, most humane care possible, and ensuring that staff are able to work in an environment that is safe and rewarding. Consequently, it is vital that interventions are designed that can deliver improvements to the social climate within acute mental health settings, when needed. Underpinning these efforts must be good quality, robust research that tests whether these interventions are indeed working to improve the climate. Achieving this requires at least two things. First, research utilizing robust evaluation design and methodology is needed (e.g., research using a pretest–posttest design, multiple baseline measurements, multiple follow-ups, and use of different and multiple scales). Second, reliable and valid measures of social climate are needed that can detect changes in climate within acute mental health inpatient settings. As highlighted by this review, such measures are currently lacking, meaning that research has often been forced to rely on questionnaires that were either not designed for use in acute mental health settings (e.g., EssenCES) or that have questionable psychometric properties (e.g., WAS). Without such measures, it will be very difficult to develop a reliable evidence base to guide clinicians and treatment managers, ultimately hindering any attempts to improve the conditions in inpatient mental health settings for both staff and patients.

## Supplemental Material

sj-docx-1-son-10.1177_23779608221124291 - Supplemental material for Interventions to Improve Social Climate in Acute Mental Health Inpatient Settings: Systematic Review of Content and Outcomes Click here for additional data file. Supplemental material, sj-docx-1-son-10.1177_23779608221124291 for Interventions to Improve Social Climate in Acute Mental Health Inpatient Settings: Systematic Review of Content and Outcomes by Geoffrey L. Dickens, Alisha Johnson, Kelly Steel, Bronwyn Everett and Matthew Tonkin in SAGE Open Nursing

sj-docx-2-son-10.1177_23779608221124291 - Supplemental material for Interventions to Improve Social Climate in Acute Mental Health Inpatient Settings: Systematic Review of Content and Outcomes Click here for additional data file. Supplemental material, sj-docx-2-son-10.1177_23779608221124291 for Interventions to Improve Social Climate in Acute Mental Health Inpatient Settings: Systematic Review of Content and Outcomes by Geoffrey L. Dickens, Alisha Johnson, Kelly Steel, Bronwyn Everett and Matthew Tonkin in SAGE Open Nursing
