# Supplementary material for: Interventions to Improve Social Climate in Acute Mental Health Inpatient Settings: Systematic Review of Content and Outcomes
Source: SAGE Open Nurs. 2022 Dec 12;8:23779608221124291. doi: 10.1177/23779608221124291 (PMC9749049; doi:10.1177/23779608221124291)
Supplement: sj-docx-1-son-10.1177_23779608221124291 - Supplemental material for Interventions to Improve Social Climate in Acute Mental Health Inpatient Settings: Systematic Review of Content and Outcomes [file sj-docx-1-son-10.1177_23779608221124291.docx]

| **Table S1: Study quality** | | | | | |  |  |  |  |  |  |  |  |  |  |  |  |
| --- | --- | --- | --- | --- | --- | --- | --- | --- | --- | --- | --- | --- | --- | --- | --- | --- | --- |
|  | Aubry et al (1996) | Baumgardt et al (2019) | Berg & Hallberg (1999) | Berry et al (2016) | Bjorkdahl (2013) | | Bowers et al (2015) | Corey (1986) | Eliassen (2016) | Frolich et al (2018) | Gartshore (2018) | Gebhardt et al (1999) | Haller et al (1996) | Hansen & Slavin (1996) | Kerfoot (2012) | Kristensen et al (2015) | Mistral (2002) |
| **A.SELECTION BIAS** |  |  |  |  |  | |  |  |  |  |  |  |  |  |  |  |  |
| 1. Representative sample | + | + | + | + | + | | + | + | + | + | + | + | + | + | +/- | + | + |
| 1. Response rate | >80% | >80% | U | <60% | U | | U | U | >80% | U | + | >60% | >80% | U | U | >60% | U |
| ***Section rating*** | S | S | M | M | M | | W | M | S | M |  | M | S | M | W | M | M |
| **B.STUDY DESIGN** |  |  |  |  |  | |  |  |  |  |  |  |  |  |  |  |  |
| 1. Type | Cohort  AB | Cohort AB | Cohort  AB | Cluster  RCT | Cohort AB | | Cluster RCT | Cohort AB | Cohort AB | Cohort AB | Cohort AB | Cohort AB | Cohort AB | Cluster trial | Cohort AB | Cohort AB | Cohort AB |
| 1. Randomized? | - | - | - | + | - | | + | - | - | - | - | - | - | - | - | - | - |
| 1. Randomization described? | NA | NA | NA | + | NA | | + | NA | NA | NA | NA | NA | NA | NA | NA | NA | NA |
| 1. Randomization appropriate | NA | NA | NA | + | NA | | + | NA | NA | NA | NA | NA | NA | NA | NA | NA | NA |
| ***Section rating*** | M | M | M | S | M | | S | M | M | M | M | M | M | M | M | M | M |
| **C.CONFOUNDERS** |  |  |  |  |  | |  |  |  |  |  |  |  |  |  |  |  |
| 1. A priori group equivalence | NA | NA | NA | + | - | | + | U | + | U | U | NA | U | NA | NA | NA | NA |
| 1. % Confounders controlled for | NA | NA | NA | NA | - | | + | U | NA | U | U | NA | - | NA | NA | NA | NA |
| ***Section rating*** | NA | NA | NA | M | W | | S | W | M | W | W | NA | W | NA | NA | NA | NA |
| **D.BLINDING** |  |  |  |  |  | |  |  |  |  |  |  |  |  |  |  |  |
| 1. Blind outcome assessor? | SR | SR | SR | SR | SR | | + | SR | SR | SR | SR | SR | SR | SR | SR | SR | SR |
| 1. Study participants blind? | U | U | U | + | U | | + | U | U | U | U | U | - | U | U | U | U |
| ***Section rating*** | W | W | W | M | W | |  | W | W | W | W | W | W | W | W | W | W |
| **E.DATA COLLECTION METHODS** | WAS | EssenCES | CCQ | WAS | E13 | |  | WAS | WAS | EssenCES | WAS | WAS‡ |  | WAS | EssenCES | SAQ | WAS |
| 1. Valid tools | + | + | + | U | +/- | | + | + | + | + | + | + | + | + | + | + | + |
| 1. Reliable tools | + | + | + | U | - | | + | + | + | + | + | + | + | + | + | + | + |
| ***Section rating*** | S | S | S | W† | W | | S | S | S | S | S | S | S | S | S | S | S |
| **F.RETENTION** |  |  |  |  |  | |  |  |  |  |  |  |  |  |  |  |  |
| 1. Withdrawals reported | NA | + | + | + | + | | - | + | + | U | + | + | + | U | U | + | + |
| 1. % completing study | U | + | U | >60% | >80% | | - |  | >60% | U | >60% | - | >60% | U | U | >60% | >80% |
| ***Section rating*** | W | S | M | M | S | | W | S | M | W | S | W | M | W | W | M | S |
| **G.INTERVENTION INTEGRITY** |  |  |  |  |  | |  |  |  |  |  |  |  |  |  |  |  |
| 1. % intervention/exposure | >80% | >80% | >80% | + | NA | | + | U | >80% | >80% | U | >80% | >80% | U | U | >60%x | >80% |
| 1. Consistency measured? | U | + | U | + | - | | + | U | U | U | U | - | + | - | - | + | - |
| 1. Unintended intervention an influence? | + | - | U | U | U | | U | - | U | U | U | + | U | + | U | - | U |
| **H. ANALYSES** |  |  |  |  |  | |  |  |  |  |  |  |  |  |  |  |  |
| 1. Unit of allocation | O | O | O | O | O | | O | O | O | O | O | O | O | O | O | O | O |
| 1. Unit of analysis | I | I | I | I | I | | I | I | P | I | I | I | I | I | I | I | I |
| 1. Appropriate statistics | + | + | + | + | + | | + | + | + | + | - | - | + | U | - | + | + |
| 1. Intention to treat analysis | - | - | - | - | - | | - | - | U | - | - | + | - | - | U | - | - |
| **I.GLOBAL RATING** | W | M | M | M | W | | M | W | M | W | W | W | W | W | W | M | M |

Table S1 continued

**Table S1 Key:** + Condition met – Condition not met +/- Condition partly met U Unclear WAS Ward Atmosphere Scale WAS* WAS rated weak in this study as amended

3-factor scale used with no details WAS ** German language version WAS*** 120-item 12-factor version EssenCES Essen Climate Evaluation Schema CCQ

Creative Climate Questionnaire W Quality rating ‘Weak’ M ‘Moderate’ S ‘Strong’; O Organisation I Institution P Practice/Office

|  | Ng et al (1982) | Nicholls et al (2015) | Pierce et al (1972) | Rigby et al (2001) | Southard et al (2012) | Thorward & Birnbaum | Urbanoski et al (2013) |
| --- | --- | --- | --- | --- | --- | --- | --- |
| **A.SELECTION BIAS** |  |  |  |  |  |  |  |
| 1. Representative sample | + | + | + | + | + | U | + |
| 1. Response rate | >80% | >60% | >80% | >60% | U | - | >60% |
| ***Section rating*** | S | M | S | M | M | W | M |
| **B.STUDY DESIGN** |  |  |  |  |  |  |  |
| 1. Type | Cohort AB | Cohort AB | Cohort AB | Cohort AB | Cohort AB | Cohort AB | Cohort^a^ |
| 1. Randomized? | - | - | - | - | - | - | - |
| 1. Randomization described? | NA | NA | NA | NA | NA | NA | NA |
| 1. Randomization appropriate | NA | NA | NA | NA | NA | NA | NA |
| ***Section rating*** | NA | M | M | M | M | NA | M |
| **C.CONFOUNDERS** |  |  |  |  |  |  |  |
| 1. A priori group equivalence | NA | - | NA | NA | - | NA | - |
| 1. % Confounders controlled for | NA | NA | NA | NA | - | NA | + |
| ***Section rating*** | NA | NA | NA | NA | W | W | M |
| **D.BLINDING** |  |  |  |  |  |  |  |
| 1. Blind outcome assessor? | SR | SR | SR | SR | SR | SR | SR |
| 1. Study participants blind? | U | U | U | + | U | - | U |
| ***Section rating*** | W | W | W | M | W | W | W |
| **E.DATA COLLECTION METHODS** | WAS | WAS | WAS§ | WAS | WAS | WAS | WAS |
| 1. Valid tools | + | + | + | + | + | + | + |
| 1. Reliable tools | + | + | + | + | + | + | + |
| ***Section rating*** | S | S | S | S | S | S | S |
| **F.RETENTION** |  |  |  |  |  |  |  |
| 1. Withdrawals reported | + | + | + | + | NA | NA | + |
| 1. % completing study | >80% | U | >40% | >60% | NA | NA | >60% |
| ***Section rating*** | S | M | W | M | NA | NA | M |
| **G.INTERVENTION INTEGRITY** |  |  |  |  |  |  |  |
| 1. % intervention/exposure | U | >80% | >80% | >80% | U | >80% | >80% |
| 1. Consistency measured? | - | - | - | - | + | - | NA |
| 1. Unintended intervention an influence? | U | - | U | - | NA | U | U |
| **H. ANALYSES** |  |  |  |  | U |  |  |
| 1. Unit of allocation | O | O | O | O | O | O | O |
| 1. Unit of analysis | I | I | I | I | I | I | I |
| 1. Appropriate statistics | + | + | - | + | + | + | + |
| 1. Intention to treat analysis | - | - | - | - | - | - | - |
| **I.GLOBAL RATING** | M | M | W | M | W | W | M |
