# Supplementary material for: Interventions to Improve Social Climate in Acute Mental Health Inpatient Settings: Systematic Review of Content and Outcomes
Source: SAGE Open Nurs. 2022 Dec 12;8:23779608221124291. doi: 10.1177/23779608221124291 (PMC9749049; doi:10.1177/23779608221124291)
Supplement: sj-docx-2-son-10.1177_23779608221124291 - Supplemental material for Interventions to Improve Social Climate in Acute Mental Health Inpatient Settings: Systematic Review of Content and Outcomes [file sj-docx-2-son-10.1177_23779608221124291.docx]

| **S2: Study complexity rating** | | | | | | | |  |
| --- | --- | --- | --- | --- | --- | --- | --- | --- |
| **Study** | **Mult-component** | **Difficulty & range** | **Multiple groups** | | **Multiple outcomes** | **Intervention flexibility** | **Complexity Global** |  |
| Aubry et al (1996) | D | D | S | | L | D | D |  |
| Baumgardt et al (2019) | D | S | S | | S | S | S |  |
| Berg & Hallberg (1999) | S | S | L | | D | S | S |  |
| Berry et al (2016) | S | S | D | | S | S | S |  |
| Bjorkdahl et al (2013) | S | S | D | | L | S | S |  |
| Bowers et al (2015) | D | S | D | | D | D | D |  |
| Corey et al (1986) | S | S | S | | L | S | S |  |
| Eliassen et al (2016) | S | S | S | | L | S | S |  |
| Frolich et al (2018) | S | S | D | | L | L | L |  |
| Gartshore (2018) | S | S | S | | L | D | S |  |
| Gebhardt & Steinert (1999) | L | L | D | | L | L | L |  |
| Haller et al (1996) | L | L | L | | L | L | L |  |
| Hansen & Slevin (1996) | S | S | S | | L | S | S |  |
| Kerfoot et al (2012) | D | D | D | | D | D | D |  |
| Kristensen et al (2015) | D | S | D | L | | S | S | |
| Mistral et al (2002) | S | S | S | S | | S | S | |
| Ng et al (1982) | L | L | S | S | | L | L | |
| Nicholls et al (2015) | S | L | S | L | | L | L | |
| Pierce et al (1972) | S | S | S | L | | S | S | |
| Rigby et al (2001) | S | L | L | S | | L | L | |
| Southard et al (2012) | L | L | L | L | | L | L | |
| Thorward & Birnbaum (1989) | L | L | L | L | | L | L | |
| Urbanoski et al (2013) | L | L | L | D | | L | L | |

L - Low complexity, S = Some complexity, D = Definite complexity. Global complexity rated:
D = 3 or more categories deemed D; S = 3 or more categories rated D or S; L = Any other
combination.
